# Comparative efficacy of aromatase inhibitors and gonadotropin-releasing hormone analogue in increasing final height of idiopathic short stature boys: a network meta-analysis

**DOI:** 10.3389/fendo.2023.1167351

**Published:** 2023-04-14

**Authors:** Siqi Wang, Zhixin Wu, Yang Chen, Kuanhong Luo, Zhenhai Cui, Jiaoyue Zhang, Juan Zheng, Kangli Xiao, Huiqing Li

**Affiliations:** Department of Endocrinology, Union Hospital, Tongji Medical College, Huazhong University of Science and Technology, Wuhan, Hubei, China

**Keywords:** idiopathic short stature, aromatase inhibitors, gonadotropin-releasing hormone analogue, final height, network meta-analysis

## Abstract

**Objective:**

To investigate the efficacy of monotherapy with AIs or GnRHa in improving the height of boys with idiopathic short stature (ISS).

**Method:**

We performed a systematic search in Pubmed, The Cochrane Library, Chinese National Knowledge Infrastructure databases, and Wanfang Database for eligible studies. The network meta-analysis was conducted using STATA software.

**Results:**

We identified a total of four studies that included 136 individuals. We used FAH/PAH as the main outcome of final height. The results revealed a statistically higher final height after treatment with AI or GnRHa in idiopathic short stature children(MD= 4.63, 95% CI[3.29,5.96]). In network meta-analysis, the direct and indirect comparison between AI and GnRHa was presented in the forest plot. Compared with control group, both AI and GnRHa were effective in increasing the final height, with the mean effect of 4.91(95%CI:1.10,8.17) and 5.55(95%CI:1.12,9.98) respectively. However, there was no statistical difference between the GnRHa and AI treatment, of which the mean effect was 0.65(95%CI: -4.30,5.60).

**Conclusion:**

Both AIs and GnRHa monotherapy were effective in augmenting the final height of boys with idiopathic short stature when compared to placebo groups. However, there was no statistical difference between the GnRHa and AI treatments.

## 1 Introduction

A common reason for consulting an endocrinologist in the clinic is concern about the final adult height (FAH) of short-statured children. It was estimated that nearly 80% of children with short stature are diagnosed with idiopathic short stature (ISS) (1). The diagnosis of idiopathic short stature (ISS) is made when an individual’s height is less than 2 standard deviations of the respective mean height for a specific age, gender, and population group, excluded from other causes such as systemic, endocrine, nutritional, or chromosomal-related diseases (2).

Aromatase inhibitors (AIs) are compounds that block the conversion of androgens to estrogens and reduce the action of estrogens at the growth plate. For this reason, AIs have been used to intervene on height outcomes in boys with GH deficiency, idiopathic short stature, constitutional growth delay, and other conditions (3–5). In ISS, AI has been found to enable ISS boys to achieve greater adult height in combination with rhGH (6, 7). As for AI monotherapy, the current studies have shown controversial results. Several studies have shown that AI monotherapy increased the growth potential of children with idiopathic short stature (8, 9). In contrast, Tero found no advantage of AI in promoting final height over placebo (10).

Gonadotropin-releasing hormone analogues (GnRHa) have been the standard treatment for central precocious puberty (CPP) since the 1980s (11). By inhibiting the secretion of gonadotrophins and gonadal steroids in children with CPP, GnRHa has been shown to decelerate bone maturation and eventually improve the FAH (12–14). However, GnRHa findings in patients with CPP are not completely consistent. GnRHa is generally thought to be effective in increasing adult height in girls younger than 6 years of age, but fails to improve final height in girls older than 8 years of age (11).In addition, in children with ISS, Khawaja’s study indicates that GnRHa monotherapy slightly increases the FAH in girls with ISS but not in boys (15). The result from Li (16)showed a positive effect of GnRHa on increasing FAH in children with ISS when compared with placebo.

Both AI and GnRHa have been used in ISS, however, there are few comparisons of these two monotherapies. The lack of direct comparison between AI and GnRHa in clinical trials makes it difficult to decide which treatment is more efficacious. In this context, a network meta-analysis provides a quantitative synthesis of the network by comparing direct and indirect evidence across clinical trials.

Therefore, we conduct a network meta-analysis to compare the effects of aromatase inhibitors and gonadotropin-releasing hormone analogue on increasing the final adult height of children with ISS. We focus on pubertal boys, since AI and GnRHa are used in pubertal children, and AI is mostly used to treat boys in clinical practice (17).

## 2 Methods

### 2.1 Information sources and search strategy

This meta-analysis was conducted and reported following the Preferred Reporting Items for Systematic reviews and Meta Analyses (PRISMA) statement (18).

We performed a systematic search in Pubmed, The Cochrane Library, Chinese National Knowledge Infrastructure databases, and Wanfang Database for studies until December 4,2022. The following key words and their corresponding synonyms were used to conduct the search: “idiopathic short stature”, “aromatase inhibitor”, and “gonadotropin-releasing hormone analogue”. The search strategy in Pubmed was: ((((((((GnRHa[Title/Abstract]) OR (GnRH analogues[Title/Abstract])) OR (GnRH-a[Title/Abstract] OR gonadotropin-releasing hormone analogue[Title/Abstract])) OR (gonadotropin-releasing hormone analogs[Title/Abstract])) OR (Triptorelin[Title/Abstract])) OR (Leuprorelin[Title/Abstract])) OR (Buserelin[Title/Abstract])) OR ((((Aromatase Inhibitors[Title/Abstract]) OR (Letrozole[Title/Abstract])) OR (Anastrozole[Title/Abstract])) OR (Testolactone[Title/Abstract]))) AND (((Short Stature[Title/Abstract]) OR (idiopathic short stature[Title/Abstract])) OR (ISS[Title/Abstract])). Additionally, the reference lists of selected articles were also scanned for any relevant study.

### 2.2 Eligibility criteria

Articles that met the following criteria would be included: (i)Study design: clinical trials, including randomized controlled trails (RCTs) and non-randomized clinical controlled trials (CCTs); (ii)Participants: boys with idiopathic short stature; (iii)Intervention: Aromatase Inhibitors or Gonadotropin-releasing hormone analogue; (iv)Comparison: other treatments or no treatment control; and (v)Outcome: final height, including final adult height (FAH) and final predicted adult height(FPAH).

### 2.3 Study selection

Based on the search strategy and inclusion criteria above, two authors searched articles and screened the titles and abstracts of them independently. Next, the two authors reviewed the full text to assess the eligibility of the selected articles. Different selections were discussed by the two authors or a third senior author was consulted to meet a consensus.

### 2.4 Data extraction and quality assessment

Data extraction from the included articles was finished by two authors independently. The following items were collected: first author, year of publication, sample size in each group, participants’ characteristics, treatment, and clinical outcomes. Clinical outcomes include predicted adult height (PAH), target height (TH), and final adult height (FAH). FAH was considered to be attained when growth was less than 1cm in a year, and/or the bone age was over 15 years (19).

The quality of included studies was assessed by two authors independently. The evaluation of the risk of bias was conducted with the tool provided in the Cochrane handbook (20).

Any disagreement during the data collection and quality assessment between the two authors was discussed or another author was consulted to find a conclusion.

### 2.5 Statistics analyses

The network meta-analysis was conducted using STATA software (version 17.0, Stata Corporation, TX, USA). The network graph and rank ordering graph of treatments were drawn by STATA. As continuous variables, the outcomes were presented as mean differences(MDs) and 95% confidence interval (CI). Heterogeneity among the studies was assessed with Chi-square and I^2^ tests. When the test result was p < 0.1 or I^2^ > 50%, the data were considered as high heterogeneity and a random effects model was used for statistics. Otherwise, a fixed effects model was adopted. In this network meta-analysis, the consistence assumption was assessed by a local inconsistence test and loop-specific test. Local inconsistency was evaluated by the node-splitting method.

## 3 Results

### 3.1 Study selection

**Figure 1:**
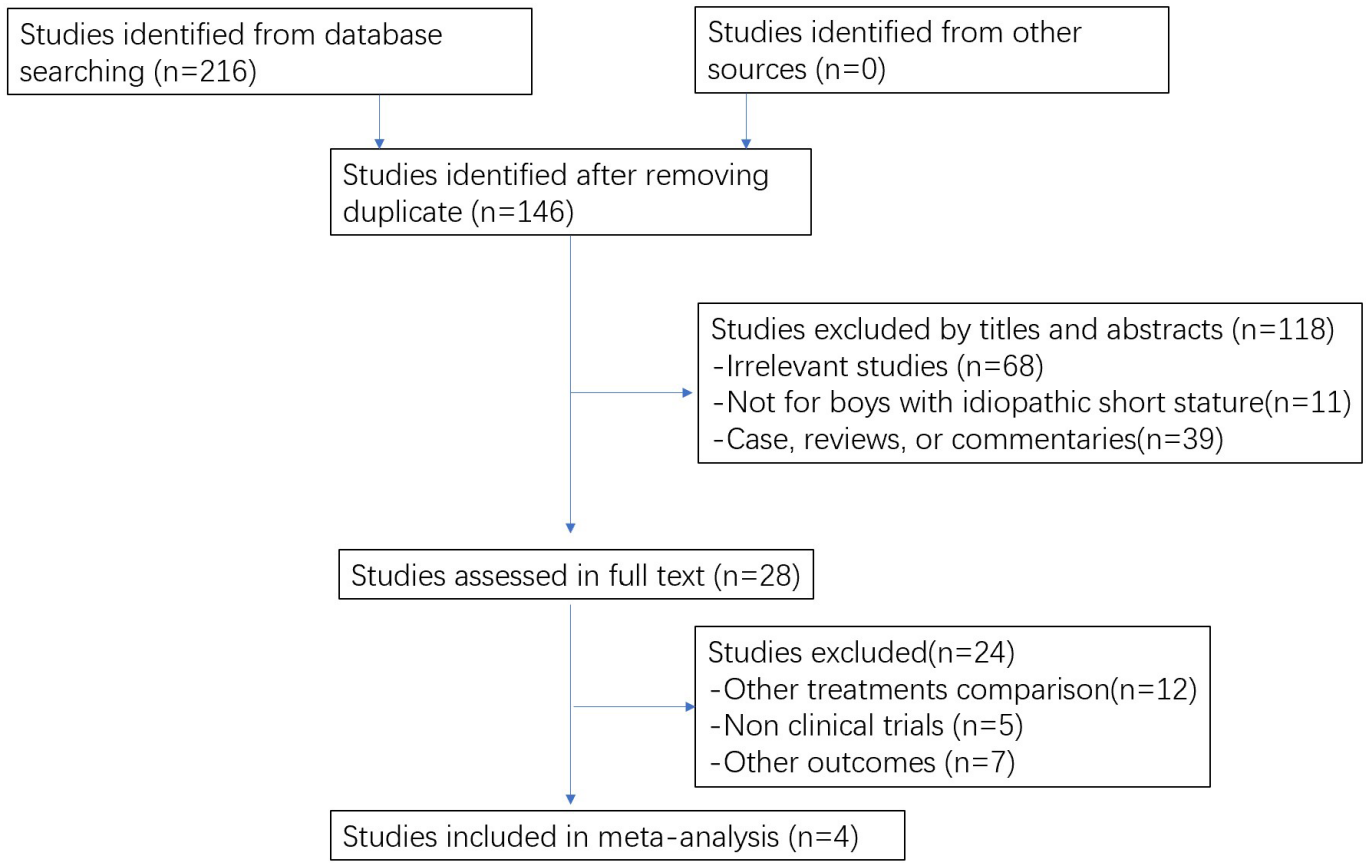
The flowchart of study selection.

In total, 216 studies were initially identified in our electronic search and 146 studies were obtained after removing duplicate studies. Following screening by title and abstract, 29 studies were obtained to evaluate in full texts. Finally, four studies met the criteria and were included in this review. The searching and selecting process is shown in **Figure 1**.

### 3.2 Study characteristics

The main characteristics of the included studies of this meta-analysis are presented in **Table 1**. The included studies were published between 2005 and 2020, covering three countries (Finland, Jordan, and China). In total, 146 male participants were included. The mean ages of these participants ranged from 10.9 to 13.8 years.

**Table 1:** Characteristics of included studies in this meta-analysis.

Study ID	Sample size	Sex	Treatment	CA(yrs)	BA(yrs)	Treatment course (yrs)	PAH(cm)	TH(cm)	FAH(cm)
Li 2020 (16)	28 30 17	male	Letrozole GnRHa No treatment	13.2 ± 0.7 12.4 ± 1.1 13.7 ± 1.4	12.9 ± 0.5 13.0 ± 0.4 13.0 ± 0.4	2.1 ± 0.7 2.3 ± 0.6 -	160.9 ± 3.3 160.2 ± 3.0 160.3 ± 3.3	168.1 ± 2.7 168.5 ± 3.2 170.0 ± 3.7	170 ± 4 170 ± 6 162 ± 4
Hero 2005 (8)	16 14	male	Letrozole No treatment	11.0 ± 1.7 11.0 ± 1.5	9.1 ± 2.3 8.9 ± 1.8	2 2	172 ± 8 166 ± 9	–	–
Tero 2019 (10)	8 9	male	Letrozole No treatment	11.5 ± 1.8 10.9 ± 1.8	9.2 ± 2.6 8.7 ± 1.9	2 2	167.6 ± 7.9 166.9 ± 3.9	–	164.8 ± 4.0 163.7 ± 3.7
Khawaja 2019 (15)	7 17	male	GnRHa No treatment	12.6 ± 1.7 13.8 ± 1.3	11.6 ± 2.7 12.6 ± 1.1	1.3 ± 0.3 1.3 ± 0.3	161.5 ± 12.2 163.8 ± 8.3	–	156.4 ± 4.7 152.3 ± 5.7

The symbol "-" means "Unavailable".

### 3.3 Quality assessment

**Figure 2:**
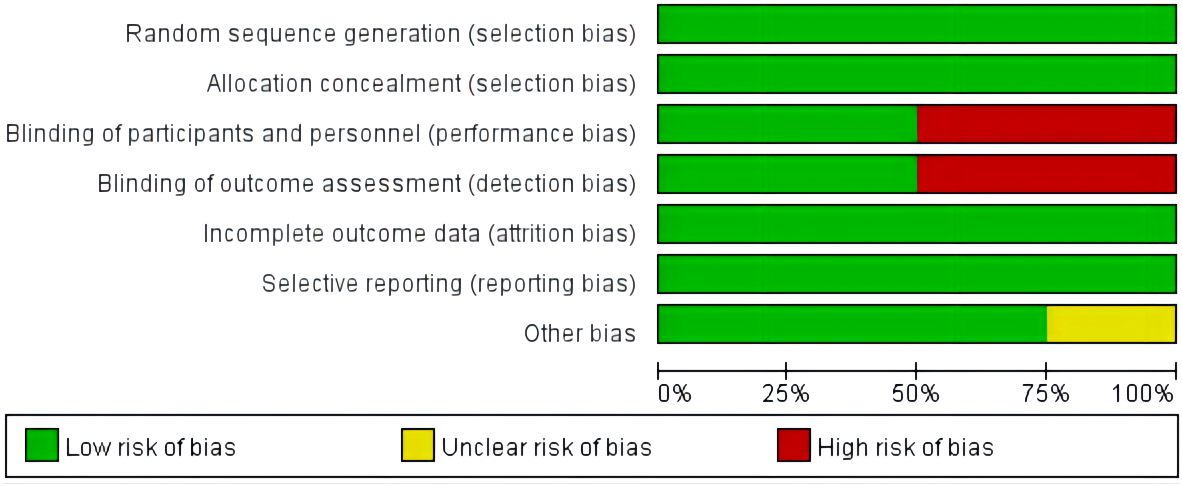
Assessment of the risk of bias in the analyzed studies. Risk of bias graph: review authors’ judgements about each risk of bias item presented as percentages across all included studies.

The quality of the included literature was evaluated using the Cochrane bias risk assessment tool. There are three levels of bias risk assessment: Low risk, Unclear risk, and high risk.These are shown in **Figure 2**.

### 3.4 Synthesis of results

#### 3.4.1 Inconsistence test

The inconsistency model was utilized to determine the inconsistency of network meta-analysis. With the result of Chi2 (2)=2.32,P= 0.3128(>0.05),it showed that the inconsistency was not significant. Thus, the fixed effects model was adopted for network meta-analysis.

Moreover, the consistence assumption was assessed by a local inconsistence test using the node-splitting method. No local inconsistency was found (p value>0.05). Next, the loop-specific test was conducted, indicating that the loop inconsistency was not significant and there was a closed loop in the network.Generally, the network was consistent with the principles of coherency, transitivity, and consistency.

#### 3.4.2 Ranking probability of treatments

**Figure 3:**
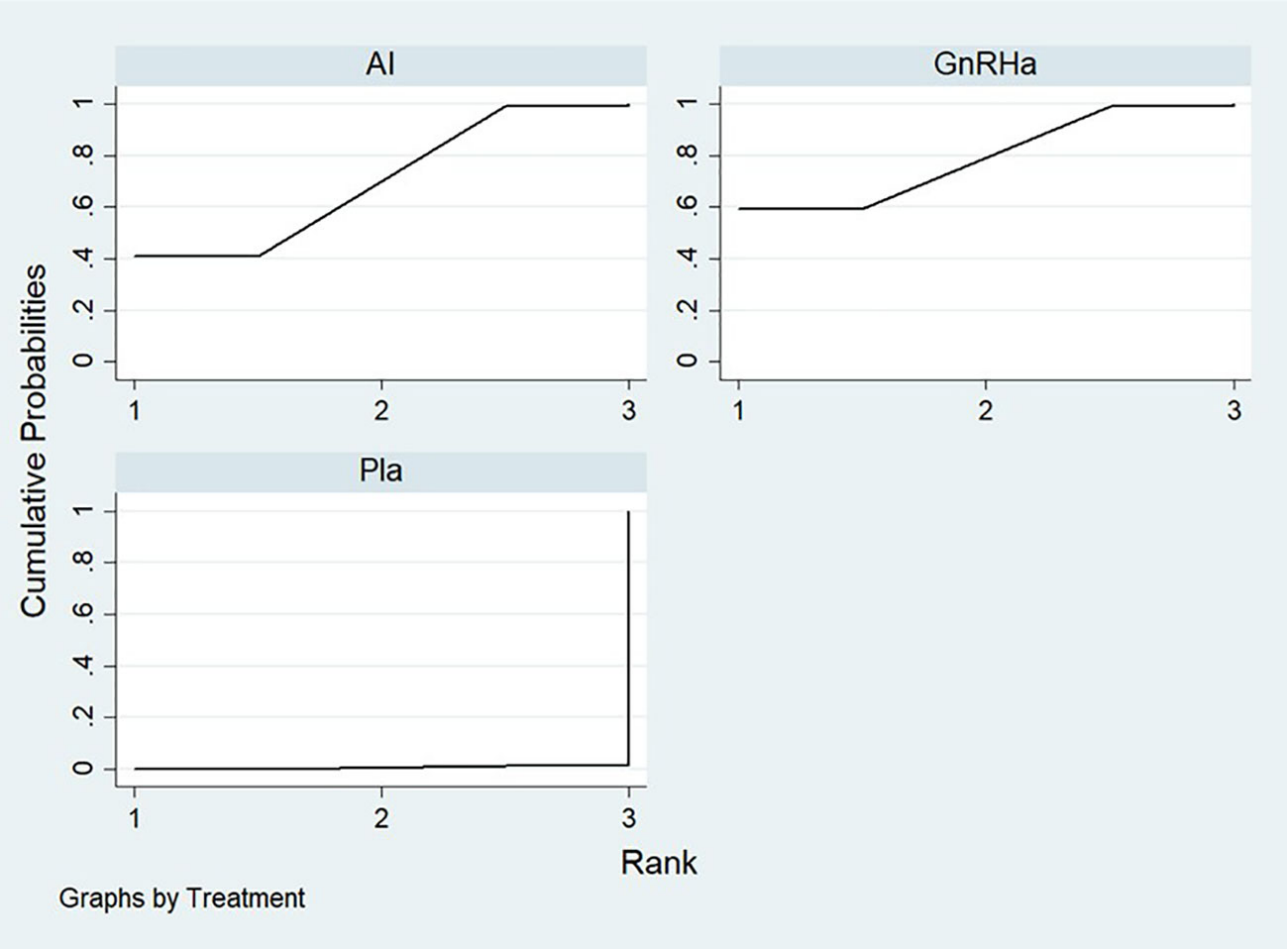
The SUCRA of placebo, AI, and GnRHa treatment.

The ranking probability of all treatments is shown in **Table 2** and **Figure 3**. The surface under the cumulative ranking curve values (SUCRA values) are used to represent the ranking probability of each treatment. The intervention with a larger SUCRA value was considered to be the more effective treatment (21).The SUCRA of placebo, AI, and GnRHa treatment are 0.6%, 70.1%, and 79.3% respectively. Covering the largest surface, GnRHa had the highest probability of being the most effective treatment.

**Table 2:** Ranking for the treatments in the network meta-analysis.

Treatment	SUCRA	PrBest	MeanRank
Placebo	0.6	0.0	3.0
AI	70.1	40.7	1.6
GnRHa	79.3	59.3	1.4

#### 3.4.3 Meta analysis

**Figure 4:**
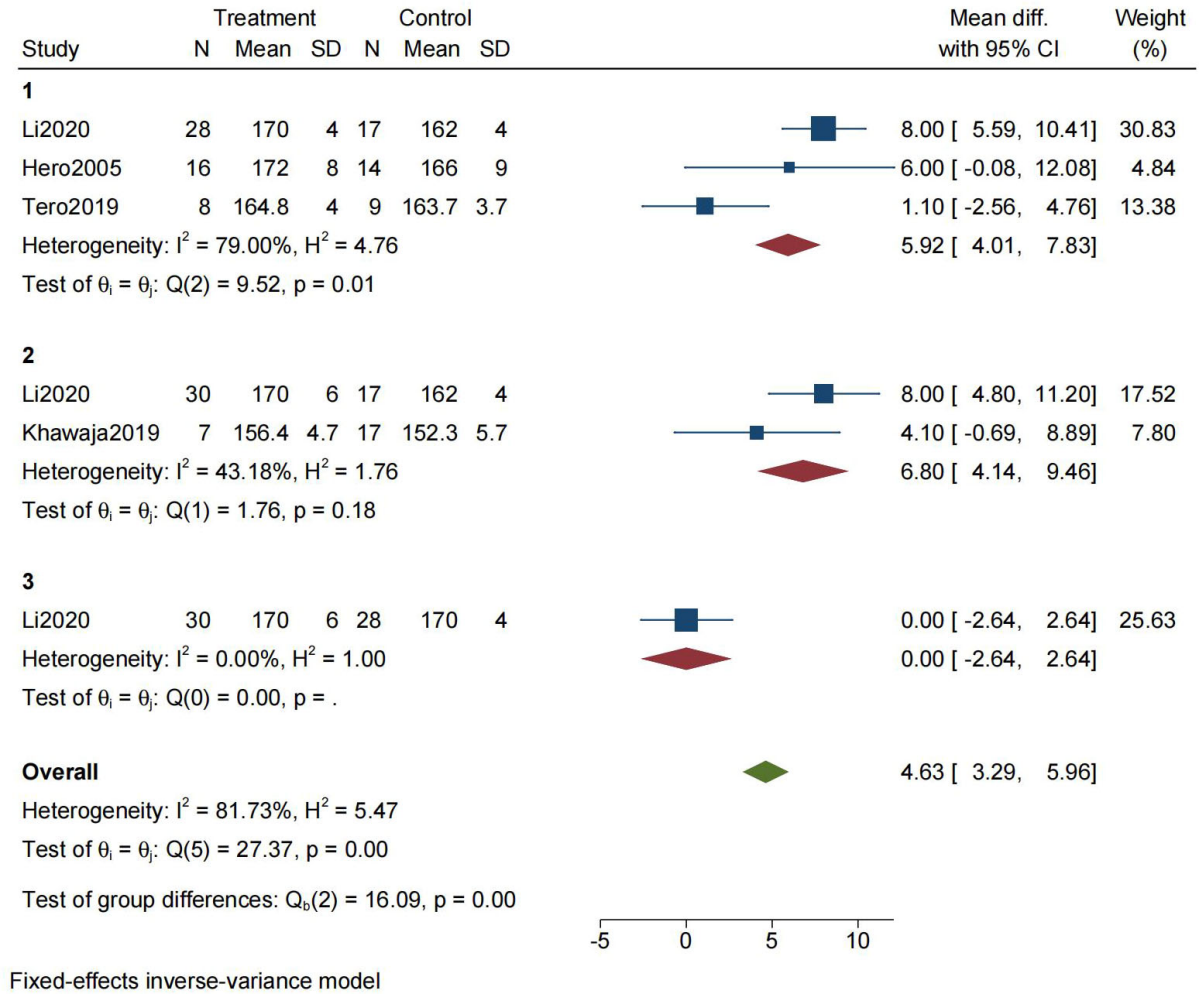
Forest plot for the meta-analysis of final height (1=AI vs No treatment, 2=GnRHa vs No treatment, 3=GnRHa vs AI).

We used FAH as the main outcome of final height. One of the included studies(Hero,2005 (8)) did not provide FAH, so PAH was used for statistic analysis instead. The results revealed a statistically higher final height after treatment with AI or GnRHa in idiopathic short stature children. (MD= 4.63, 95% CI[3.29,5.96])(**Figure 4**).

**Figure 5:**
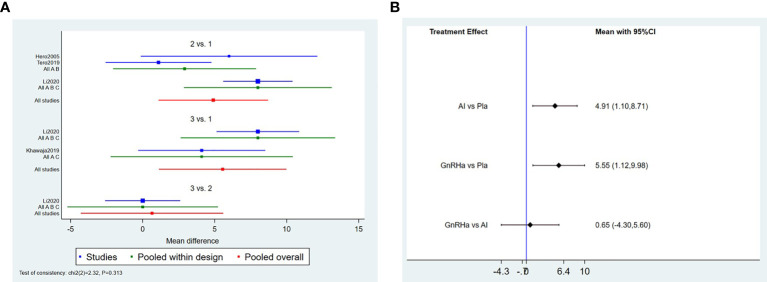
Forest plot of comparisons among treatments.**(A)** Forest plot for the network meta-analysis of treatment with AI and GnRHa. **(B)** Forest plot for the pairwise comparsion of the different treatments.

In network meta-analysis, the direct and indirect comparison between AI and GnRHa was presented in the forest plot (**Figure 5**; **Table 3**). Compared with the control group, both AI and GnRHa were effective in increasing the final height, with the mean effect of 4.91(95%CI:1.10,8.17) and 5.55(95%CI:1.12,9.98) respectively. However, there was no statistical difference between the GnRHa and AI treatment, of which the mean effect was 0.65(95%CI: -4.30,5.60).

**Table 3:** Result of comparisons among treatments.

GnRHa		
0.65 (-4.30,5.60)	AI	
5.55 (1.12,9.98)	4.91 (1.10,8.71)	Placebo

## 4 Discussion

The purpose of this meta-analysis was to evaluate the efficacy of monotherapy with AIs or GnRHa in improving the height of boys with ISS. The crucial findings of this study were that both AIs and GnRHa monotherapy were effective in augmenting the final height of boys with idiopathic short stature when compared to placebo groups. We further compared the effectiveness of AIs and GnRHa; the results showed that the boys who were treated with AIs had similar final heights with those who received GnRHa.

To delay skeletal maturation and gain extra height, the use of AIs falls into two main groups of conditions: precocious puberty and idiopathic short stature (22). Previous studies have shown AI therapy can effectively improve final height in idiopathic short children. In a randomized controlled trial, the authors found that PAH changed significantly in the AI-treated group compared to the placebo and untreated group (The increase was on average 5.1 cm) (5). However, no FAH data were reported. In Hero’s study, the boys with constitutional delay of growth and puberty (CDGP) treated with testolactone and AI reached a higher mean near-final adult height than did boys on testolactone and placebo (175.8cm vs. 169.1cm; P = 0·04) (23). The first study in evaluating the effect of AI (letrozole) on FAH in boys with CDGP showed that the FAH for the AI group were significantly higher than that of the control group (-171 ± 4.5cm vs. 168.8 ± 4.1cm; P=0.04) (24). Another case report on the 5-year AI impact of ISS boys demonstrated that FAH was 15cm higher than PAH before treatment (25). In contrast, Tero’s study found that AI monotherapy did not improve the patients’ FAH (10). The results of our meta-analysis are consistent with most studies. Compared with the control group, AI was effective in increasing the final height, with the mean effect of 4.91(95%CI:1.10,8.17), which indicates that AI may improve FAH of ISS in boys. One possible explanation for the inconsistency in these research findings is insufficient sample size. Thus, larger placebo-controlled RCTs, targeted to idiopathic short children, are needed to assess the efficacy of AI therapy on improving adult height.

The efficacy of gonadotropin-releasing hormone agonist (GnRHa) is undisputed in improving adult stature in girls presenting with CPP up to age 6, but its efficacy is not yet clear for older age groups and boys (11). At present, controversial literature exists concerning the effectiveness of GnRHa on treating ISS children. A clinical historical cohort study was carried out on 28 children with ISS who received GnRHa treatment (21 girls and seven boys) and 31 controls with ISS (14 girls and 17 boys). The results revealed that GnRHa therapy has a modest effect in improving FAH in girls with ISS (151.3 ± 5.1 vs. 146.8 ± 3.8 cm; p = 0.01), but not in boys (156.4 ± 4.7cm vs. 152.3 ± 5.7cm; p = 0.111) (15). In addition, three other studies showed no effect in girls (26–28). Unfortunately, the research in boys is limited to these studies. In Li’s study, adolescent male children with ISS were found to improve their adult height with GnRHa treatment. Compared to the untreated group, long-term treatment with GnRHa on 30 boys effectively slowed bone age growth, resulting in an improvement in FAH (170 ± 6cm vs. 162 ± 4cm; p=0.01) (16). In our meta-analysis, a significant increase in FAH was observed in boys treated with GnRHa monotherapy. GnRHa was effective in increasing the final height with a mean effect of 5.55(95%CI:1.12,9.98). This finding was not consistent with findings from some studies on girls with ISS, which may indicate that gender is an important factor influencing efficacy.

Studies showed that AI or GnRHa monotherapy may improve the FAH in children with ISS. However, there are few studies on the differences between the efficacy of the two compounds in children with ISS. The available data suggest that aromatase inhibitors improved short‐term growth outcomes, which is better than GnRHa alone (29, 30). In order to gain an overall evaluation, we further explored the differences between the two therapies in the studies we included. It is worth noting that there was no statistical difference between the area of SUCRA of GnRHa and AI treatment, although there was a larger area under SUCRA in the GnRHa treatment group. Our study suggested that the two compounds have similar effects in improving FAH. As far as we know, this is to date the first meta-analysis to evaluate the efficacy difference between AI and GnRHa monotherapy in children with ISS.

GnRHas treatment are considered safe (29). GnRHa may affect short-term bone mineral density (BMD) (31), although this seems to be transient (32), and can cause the psychological problem of delaying puberty (33). The long-term safety of aromatase inhibitors in male patients with ISS has not been proven. Previous studies showed that Vertebral morphology was adversely affected. Transient HDL reduction, BMD reduction, and slight insulin sensitivity increase may also occur during AI treatment (17, 32). Therefore, there is more uncertainty about the long-term safety of aromatase inhibitors (34). In our study, we found that either AI or GnRHa can help improve the FAH of ISS in boys. In terms of price and convenicence, AI is preferable, whereas for long-term security, GnRHa is the better choice.

Publication bias in this study was not assessed because the number of included studies was small and the power of funnel plot asymmetry test too low to distinguish real bias. The inconsistence test was conducted, and the results indicated that the results of the direct and indirect therapy comparison for ISS patients were consistent, and PAH/FAH indexes across the included literatures were reliable.

There are several possible limitations to our meta-analysis. First, this study was aimed at the increase of final adult height. The key indices, such as the difference of final adult height SDS, the difference of final height minus PAH (FH-PAH), and the difference of final height minus TH (FH-TH), have not been comprehensively assessed (35). As a result, the conclusions were limited. Second, the population in this study consisted of boys with idiopathic short stature. Further evidence is needed to determine whether the findings can be applied to other short stature conditions and female patients. Third, the number of studies included in each therapy was limited and the sample sizes were small. Furthermore, because relevant studies were insufficient and not every trial documented final adult height, predicted adult height of Hero’s study was used instead for analysis. We have conducted an analysis exluding Hero’s study, nevertheless, and it shows that no heterogeneous source was found for those three papers. Since it is difficult to conduct statistical analysis if that study is excluded, we decided to include it in our research. The above limitation could affect the reliability of the final outcome.Therefore, more high-quality placebo-controlled RCTs and multicenter studies are required in the future to provide greater support for the clinical evidence.

## 5 Conclusion

Compared with no treatment, the current evidence indicates that both AI and GnRHa treatment improve the FAH of boys with ISS. However, the sample sizes were small, so more studies are needed to confirm the long-term safety of AI.

## Data availability statement

The original contributions presented in the study are included in the article/supplementary material. Further inquiries can be directed to the corresponding authors.

## Author contributions

HL: protocol development, manuscript review, and revision; KX: data analysis and manuscript revision; SW: study selection, data collection, and partial drafting; ZW: study selection, data collection, and partial drafting; YC: data analysis and partial drafting; KL: literature search and quality assessment; ZC: literature search and quality assessment; JYZ: data interpretation; JZ: data interpretation. All authors have read and approved the final manuscript.
